# The impact of the Nutri-Score front-of-pack nutrition label on purchasing intentions of unprocessed and processed foods: post-hoc analyses from three randomized controlled trials

**DOI:** 10.1186/s12966-021-01108-9

**Published:** 2021-03-17

**Authors:** Manon Egnell, Pilar Galan, Morgane Fialon, Mathilde Touvier, Sandrine Péneau, Emmanuelle Kesse-Guyot, Serge Hercberg, Chantal Julia

**Affiliations:** 1 Sorbonne Paris Nord University, Inserm U1153, Inrae U1125, Cnam, Nutritional Epidemiology Research Team (EREN), Epidemiology and Statistics Research Center – University of Paris (CRESS), SMBH Paris 13, 74 rue Marcel Cachin, F-93017 Bobigny Cedex, France; 2 Public Health department, Avicenne Hospital, Assistance Publique des Hôpitaux de Paris (AP-HP), Bobigny, France

**Keywords:** Front-of-pack nutrition label, Purchasing behavior, Pre-packed foods, processed foods

## Abstract

**Background:**

The Nutri-Score summary graded front-of-pack nutrition label has been identified as an efficient tool to increase the nutritional quality of pre-packed food purchases. However, no study has been conducted to investigate the effect of the Nutri-Score on the shopping cart composition, considering the type of foods. The present paper aims to investigate the effect of the Nutri-Score on the type of food purchases, in terms of the relative contribution of unpacked and pre-packed foods, or the processing degree of foods.

**Methods:**

Between September 2016 and April 2017, three consecutive randomized controlled trials were conducted in three specific populations – students (*N* = 1866), low-income individuals (*N* = 336) and subjects suffering from cardiometabolic diseases (*N* = 1180) – to investigate the effect of the Nutri-Score on purchasing intentions compared to the Reference Intakes and no label. Using these combined data, the proportion of unpacked products in the shopping carts, as well as the distribution of products across food categories taking into account the degree of processing (NOVA classification) were assessed by trials arm.

**Results:**

The shopping carts of participants simulating purchases with the Nutri-Score affixed on pre-packed foods contained higher proportion of unpacked products – especially raw fruits and meats, i.e. with no FoPL –, compared to participants purchasing with no label (difference of 5.93 percentage points [3.88–7.99], *p*-value< 0.0001) or with the Reference Intakes (difference of 5.27[3.25–7.29], p-value< 0.0001). This higher proportion was partly explained by fewer purchases of pre-packed processed and ultra-processed products overall in the Nutri-Score group.

**Conclusions:**

These findings provide new insights on the positive effect of the Nutri-Score, which appears to decrease purchases in processed products resulting in higher proportions of unprocessed and unpacked foods, in line with public health recommendations.

**Supplementary Information:**

The online version contains supplementary material available at 10.1186/s12966-021-01108-9.

## Introduction

In order to prevent nutrition-related chronic diseases, Front-of-Pack nutrition Labels (FoPL) have been identified as efficient tools to encourage consumers towards healthier food choices [1–3] and to promote food reformulation [4, 5]. Multiple formats have been implemented worldwide, including purely informative or interpretive schemes. In France, the Reference Intakes (RIs) have been implemented by some food manufacturers in 2006, providing numerical information on the nutritional content of foods. Currently, they co-exist on the French market with the Nutri-Score, a summary graded color-coded FoPL officially adopted in October 2017 by the French public health authorities to be applied on pre-packed foods – on a voluntary basis given the European regulation [6] – and then implemented in multiple European countries, including Spain, Belgium, Netherlands, Luxembourg and Switzerland. FoPLs have been shown to improve the nutritional quality of food purchases [7–18]. Nevertheless, concerns have been expressed by experts on some potential disadvantages of FoPLs [19]. The implementation of FoPLs on pre-packed foods only may notably influence consumers’ choices towards processed foods carrying a favourable label rather than unprocessed products such as fresh fruits and vegetables which do not carry a label as unpacked [19] through a halo effect, while public health recommendations encourage the consumption of unprocessed foods [20]. However, to our knowledge no study has investigated the influence of the Nutri-Score and other FoPLs on the composition of food purchases, concerning the degree of processing of the products in the shopping cart. While the Nutri-Score calculation is based on the nutritional quality of foods only without considering the processing dimension, it would be interesting to test the indirect impact of this FoPL on purchases in terms of food processing degree. The present study aims to assess the effect of the Nutri-Score on food purchasing intentions concerning the type food (unpacked versus pre-packed) and the processing degree of the products in the shopping carts (i.e. unprocessed, processed or ultra-processed food products). For this purpose, data from three randomized controlled trials, aiming to investigate the impact of the Nutri-Score, compared to the current labelling situation in France (i.e., Reference Intakes (RIs) or no label), on food purchasing intentions of, were used [21]. The effect was investigated among populations at-risk of having less healthy diets (i.e. students, low-income population), or for which an improvement of dietary intakes is part of a treatment strategy (i.e. individuals suffering from cardiometabolic diseases).

## Subjects and methods

Between September 2016 and April 2017, three-arm parallel group randomized controlled trials (https://clinicaltrials.gov/ct2/show/NCT02769455) were conducted sequentially using an experimental online supermarket and including individuals engaged in grocery shopping. The design of the first trial among students and the recruitment have been described elsewhere [21]. Briefly, the first trial included students from multiple French universities, aged between 18 and 25 years old. The second trial included active adults from the French NutriNet-Santé cohort [22], aged between 30 and 50 years old, and having a monthly income below 1200€ per consumption unit (i.e. corresponding roughly to the second decile of income). The third trial focused on individuals over 50 years old within the NutriNet-Santé cohort also, suffering from at least one nutrition-related cardiometabolic diseases (obesity, type 2 diabetes, dyslipidaemia, arterial hypertension, cardiovascular disease). The three trials were conducted following the same protocol as the trial on students and differed only on the targeted population [21]. Participants were invited to fulfill an inclusion questionnaire to collect data on the eligibility criteria mentioned above, as well as information on various sociodemographic characteristics and nutrition-related behaviors. Eligible participants were then randomly allocated to one of the three arms using a random block method, and invited to simulate a shopping situation as if they were in their usual supermarket. For the three trials, the final sample size was calculated considering an effect size of 0.2 (for the main outcome, the FSAm-NPS score of the shopping cart), a power of 90% and a *p*-value of 0.02 considering the three-arm design, resulting in 1956 individuals, i.e. 652 participants per arm (**Supplemental Figure 1** in Additional file 1). To reach this final sample size while considering the non-respondent rate, the number of participants validating their shopping cart was monitored.

In the experimental arm of the three trials, the Nutri-Score was affixed on the front of the package of all pre-packed foods in the online supermarket – no label was applied on unpacked foods (e.g., fresh fruits and vegetables, butcher meat). Briefly, the Nutri-Score is a summary graded scale indicating the overall nutritional quality of a food product, based on the United Kingdom Food Standards Agency Nutrient Profiling System, adapted to the French context by the High Council for Public Health (FSAm-NPS) [23]. Considering the nutritional content of food in unfavorable (energy, Saturated Fatty Acids (SFA), sugars, sodium) and favorable elements (proteins, fibers, fruits, vegetables, legumes and nuts), the FSAm-NPS score ranged from − 15 points for products with higher nutritional quality to + 40 points for foods with lower nutritional quality. The Nutri-Score is then calculated using the FSAm-NPS score and expressed through a graded scale between “A” in dark green for healthier products (e.g. fresh fruits, vegetables, whole-grain bread) and “E” in dark orange for unhealthier products (e.g. processed meat, butter, chocolate or cookies). In the three trials, two control arms were included: (i) no label, and (ii) the RIs affixed on the front of pre-packed foods. The RIs label is a nutrient-specific FoPL providing the content in energy, fats, SFA, sugars and sodium in gram per serving, as well as their percentage contribution to the guideline-based daily intakes [24]. The two schemes that were tested are displayed in **Supplemental Figure 2** (Additional file 2).

The experimental online supermarket was created to resemble existing online supermarket and allowed participants to simulate a purchasing situation, though without any payment. The supermarket included 751 different foods and beverages, with raw and unpacked fresh products (*N* = 56 foods) and pre-packed foods (*N* = 695 foods). Selection of products was made in order to reproduce the food offer available in online grocery stores. Information on the nutritional composition, ingredients list, and the price of the product was provided for all foods on the experimental supermarket, with in addition the FoPL on pre-packed foods in the Nutri-Score and RIs arms. The products from the experimental online supermarket were classified in 36 food categories, including four categories containing foods with various degrees of processing. Therefore, for the categories of “fruits”, “vegetables”, “meat” and “fish”, products were categorized according to their degree of processing, using the NOVA classification which is based on the extent and purpose of industrial food processing [25]. The NOVA classification categorizes foods into four groups: the group 1 includes products with no or little processing, the group 2 gathers processed culinary ingredients (e.g., sugars, oils, butter), the group 3 includes processed products (i.e. foods containing usually two or three ingredients, and which have been transformed through various methods of preservation or cooking), and the group 4 contains ultra-processed foods, for which specific industrial processes were applied (i.e. hydrogenation, hydrolises, extruding, moulding, etc) or some substances added (i.e., flavoring agents or food additives such as colors, emulsifiers, humectants, non-sugar sweeteners). In the present study, the four food categories for which products were classified according to the degree of processing did not contain any foods from NOVA group 2. Distribution showed an overall balanced distribution (33–33-34% split) across NOVA groups 1, 3 and 4 for fruit and vegetables (NOVA 1 fresh fruits and vegetables, NOVA 3 fruit purées, canned vegetables, NOVA 4 prepared fruit and vegetables with additives); a 17–29-54% split for fish (NOVA 1 fresh fish, NOVA 3 canned fish, NOVA 4 prepared fish or fish patties) and a 40–60% split between NOVA 1 and 4 for meat (NOVA 1 fresh meat cuts, NOVA 4 processed meat).

Main socio-demographic characteristics of participants collected at inclusion were described according to each of the studies performed (i.e. students, working adults with low incomes, subjects with chronic diseases). The mean proportion of unpacked and pre-packed products that were purchased in each arm was calculated. For pre-packed foods, products were distinguished according to their Nutri-Score class. Then, in each trial arm, the distribution of foods across the different food categories, considering additionally the degree of processing (NOVA group) for the four categories mentioned above, was calculated and expressed with mean proportions and standard deviations. The mean proportions were then compared overall between arms using one-way ANOVA. Then, the two-by-two differences between arms were calculated and pairwise comparisons were performed using Tukey tests to consider multiple comparisons. Analyses were conducted on the overall sample with an adjustment for the population, using the SAS software (version 9.4; SAS Institute, Inc). Tests were two-sided and a *p*-value below 0.05 was considered statistically significant.

## Results

Socio-demographic characteristics of the samples are presented in Table 1**.** Overall, the participants were predominantly female (up to 86.6% of participants for the study in working adults with low incomes) and with a high participation of highly educated participants – except notably in the study in low-income groups. Participants were the main grocery shopper for their households and were asked to perform grocery shopping online. Notably, a large proportion of participants considered that they had intermediate to low nutritional knowledge, and most reported reading the nutrition facts table infrequently.

**Table 1:** Socio-demographic characteristics of participants included in each study (*N* = 3382 participants overall)

	Students	Working adults with low incomes	Subjects with chronic diseases
**Total (N)**	1866	336	1180
**Sex, n(%)**			
*Men*	497 (26.6)	45 (13.4)	407 (34.5)
*Women*	1369 (73.4)	291 (86.6)	773 (65.5)
**Age, years, M (SD)**	20.4 ± 1.9	41.3 ± 5.9	65.0 ± 7.1
**Educational level**			
*Up to secondary education*	0	56 (16.7)	0
*Secondary education*	0	77 (22.9)	0
*Undergraduate*	1118 (59.9)	138 (41.1)	328 (27.8)
*Graduate studies*	736 (39.5)	64 (19.0)	852 (72.2)
*Others*	12 (0.6)	0	0
*Missing data*	0	1 (0.3)	0
**Grocery shopping frequency, n(%)**			
*Always*	908 (48.7)	223 (66.4)	722 (61.2)
*Often*	531 (28.4)	90 (26.8)	342 (29.0)
*Sometimes*	427 (22.9)	23 (6.8)	116 (9.8)
**Online grocery shopping, yes n(%)**	1286 (68.9)	135 (40.2)	821 (69.6)
**Perceived nutritional knowledge, n(%)**			
*High*	141 (7.6)	25 (7.4)	98 (8.3)
*Intermediate*	765 (41.0)	190 (56.6)	675 (57.2)
*Low*	863 (46.2)	110 (32.7)	374 (31.7)
*No*	97 (5.2)	11 (3.3)	25 (2.1)
*Missing data*	0	0	8 (0.7)
**Nutrition facts reading frequency, n(%)**			
*Always*	227 (12.2)	51 (15.2)	172 (14.6)
*Often*	662 (35.5)	157 (46.7)	607 (51.4)
*Sometimes*	751 (40.2)	112 (33.3)	358 (30.3)
*Never*	226 (12.1)	16 (4.8)	35 (3.0)
*Missing data*	0	0	8 (0.7)

For each trial arm, the numbers and proportions of unpacked and pre-packed products from the different classes of Nutri-Score in the shopping carts are presented in Table 2. In the Nutri-Score arm, participants tended to purchase less food products in number, compared to the RIs and no label arms, and especially less pre-packed products. Indeed, − 4.27 pre-packed products on average were purchased in the Nutri-Score group compared to no label, and − 5.82 compared to the RIs. In particular, the Nutri-Score application led to higher decrease of less healthy foods classified from ‘C’ to ‘E’. Indeed, − 1,99 product classified as ‘A’ or ‘B’ and − 2,28 products classified as ‘C’, ‘D’, or ‘E’ were purchased on average in the Nutri-Score group compared to no label, and − 2,68 products classified as ‘A’ or ‘B’ and − 3,14 products classified as ‘C’, ‘D’, or ‘E’ were purchased on average in the Nutri-Score group compared to the RIs. The proportion of unpacked food products in the shopping carts was higher in the Nutri-Score group compared to no label (difference of 5.93 percentage points [3.88–7.99], *p*-value< 0.0001) and the Reference Intakes (difference of 5.27 percentage points [3.25–7.29], p-value< 0.0001). No significant difference was observed between the Reference Intakes and no label.

**Table 2:** Numbers and proportions of unpacked and pre-packed labeled food products purchased in the three arms (*N* = 3382 participants overall)

Type of products	No label	Reference Intakes	Nutri-Score	P	Nutri-Score vs no label		Nutri-Score vs Reference Intakes		Reference Intakes vs no label	
					Difference [95%CI]	P	Difference [95%CI]	P	Difference[95%CI]	P
**Number of products**										
**Pre-packed foods**^**a**^										
Nutri-Score A^b^	7.56 ± 8.12	7.97 ± 6.63	6.03 ± 6.14	**< 0.0001**	−1.53 (−2.22;-0.84)	**< 0.0001**	− 1.94 (− 2.62;-1.26)	**< 0.0001**	0.41 (− 0.27;1.09)	0.3
Nutri-Score B^b^	3.02 ± 4.04	3.30 ± 4.08	2.56 ± 3.94	**< 0.0001**	−0.46 (− 0.86;-0.07)	**0.01**	− 0.74 (− 1.13;-0.35)	**< 0.0001**	0.28 (− 0.12;0.67)	0.2
Nutri-Score C^b^	3.55 ± 3.84	3.83 ± 4.06	3.05 ± 3.42	**< 0.0001**	−0.50 (− 0.87;-0.13)	**0.004**	− 0.78 (− 1.14;-0.41)	**< 0.0001**	0.28 (− 0.09;0.65)	0.2
Nutri-Score D^b^	5.03 ± 5.96	5.36 ± 5.21	3.66 ± 4.45	**< 0.0001**	−1.37 (− 1.88;-0.86)	**< 0.0001**	−1.70 (−2.21;-1.19)	**< 0.0001**	0.33 (− 0.18;0.84)	0.3
Nutri-Score E^b^	1.59 ± 2.30	1.84 ± 2.72	1.18 ± 1.98	**< 0.0001**	−0.41 (− 0.64;-0.18)	**< 0.0001**	−0.66 (− 0.89;-0.43)	**< 0.0001**	0.25 (0.02;0.49)	**0.03**
**Unpacked foods**^**c**^										
No label	8.08 ± 10.09	8.31 ± 6.84	6.18 ± 5.46	**< 0.0001**	−1.90 (− 2.64;-1.16)	**< 0.0001**	− 2.13 (− 2.85;-1.4)	**< 0.0001**	0.23 (− 0.51;0.96)	0.6
**Proportions**										
**Pre-packed foods**^**a**^										
Nutri-Score A^b^	27.09 ± 16.66	26.79 ± 15.35	24.39 ± 17.07	**< 0.0001**	−2.71 (−4.33;-1.08)	**0.0003**	−2.40 (− 4.00;-0.80)	**0.001**	−0.30 (−1.92;1.31)	0.9
Nutri-Score B^b^	10.65 ± 12.16	9.85 ± 9.06	8.79 ± 9.21	**< 0.0001**	−1.87 (−2.88;-0.86)	**< 0.0001**	− 1.06 (− 2.06;-0.07)	**0.04**	−0.81 (− 1.81;0.20)	0.1
Nutri-Score C^b^	12.49 ± 11.69	12.10 ± 10.57	14.91 ± 16.63	**< 0.0001**	2.43 (1.12;3.74)	**< 0.0001**	2.81 (1.52;4.10)	**< 0.0001**	−0.38 (− 1.69;0.92)	0.7
Nutri-Score D^b^	16.89 ± 12.35	16.96 ± 12.63	13.56 ± 12.36	**< 0.0001**	−3.33 (−4.57;-2.10)	**< 0.0001**	− 3.40 (− 4.61;-2.18)	**< 0.0001**	0.06 (− 1.17;1.29)	1.0
Nutri-Score E^b^	5.18 ± 6.96	5.95 ± 9.41	4.73 ± 8.33	**0.002**	−0.45 (− 1.28;0.37)	0.4	−1.22 (−2.03;-0.41)	**0.002**	0.77 (−0.05;1.59)	0.09
**Unpacked foods**^**c**^										
No label	27.69 ± 17.75	28.36 ± 17.54	33.63 ± 26.70	**< 0.0001**	5.93 (3.88;7.99)	**< 0.0001**	5.27 (3.25;7.29)	**< 0.0001**	0.66 (−1.38;2.70)	0.6

^a^ Pre-packed foods were categorized with the Nutri-Score. These products were the only foods from the experimental online supermarket carrying a Nutri-Score label given the European regulation

^b^ Nutri-Score classifies products in 5 categories of overall nutritional quality between A healthier to E less healthy

^c^ Unpacked foods included all food products from the experimental online supermarket, that were unpacked and therefore non-labeled given the European regulation

Values correspond to mean ± standard deviation

The distribution of foods across the different categories for the overall sample – considering the processing degree for some food categories – is described in Tables 3 and 4. In the Nutri-Score group, participants tended to purchase less manufactured processed or ultra-processed products, especially cheeses, delicatessen, ultra-processed fish, sugary products and salty products, sauces and condiments, and more processed fruits and seafood products, compared to no label or the RIs (Table 3). Therefore, the shopping carts of the participants exposed to the Nutri-Score contained in proportion more fruits based products, especially unprocessed and processed fruits, more unprocessed meat and to a lower extent ultra-processed meat, more fruit juices and dessert creams, and less unprocessed vegetables, legumes, starches, milk products, cheeses, delicatessen, processed and ultra-processed fishes, sweet biscuits and confectionaries, savoury aperitif products, soups, and beverages as chocolate, tea and coffee, compared to no label or the RIs (Table 4).

**Table 3:** Number of food products in the different categories by trial arm in the overall sample (*N* = 3382 participants overall)

Food category	Number of products				Nutri-Score vs no label			Nutri-Score vs Reference Intakes			Reference Intakes vs no label		
	No label	Reference Intakes	Nutri-Score	P-value	Relative difference	Difference [95%CI]	P-value	Relative difference	Difference [95%CI]	P-value	Relative difference	Difference [95%CI]	P-value
**Fruits, vegetables, legumes and nuts**													
Raw fruits	1.74 ± 2.24	1.68 ± 1.49	1.69 ± 1.61	0.8	−2.96%	−0.05 (− 0.22;0.13)	0.8	0.59%	0.01 (− 0.16;0.19)	1.0	−3.57%	−0.06 (− 0.24;0.11)	0.8
Processed fruits	0.16 ± 0.52	0.21 ± 0.55	0.24 ± 0.58	**0.003**	33.33%	0.08 (0.03;0.13)	**0.002**	12.5%	0.03 (−0.02;0.09)	0.3	23.81%	0.05 (− 0.01;0.10)	0.1
Ultra-processed fruits	0.17 ± 0.63	0.19 ± 0.51	0.16 ± 0.49	0.4	−6.25%	−0.01 (− 0.07;0.04)	0.8	−18.75%	− 0.03 (− 0.09;0.02)	0.3	10.53%	0.02 (− 0.04;0.07)	0.7
Raw vegetables	2.52 ± 3.87	2.54 ± 2.39	1.48 ± 1.80	**< 0.0001**	−70.27%	−1.04 (− 1.31;-0.76)	**< 0.0001**	−71.62%	−1.06 (− 1.33;-0.79)	**< 0.0001**	0.79%	0.02 (− 0.25;0.30)	0.9
Processed vegetables	0.63 ± 1.60	0.66 ± 1.09	0.55 ± 1.14	0.08	−14.55%	−0.08 (− 0.21;0.04)	0.2	−20%	− 0.11 (− 0.24;0.01)	0.08	4.55%	0.03 (− 0.10;0.16)	0.9
Ultra-processed vegetables	0.56 ± 1.29	0.63 ± 1.13	0.46 ± 0.80	**0.001**	−21.74%	−0.10 (− 0.21;0.01)	0.07	−36.96%	− 0.17 (− 0.27;-0.06)	**0.0007**	11.11%	0.07 (− 0.04;0.17)	0.3
Legumes	0.41 ± 0.86	0.46 ± 0.92	0.28 ± 0.70	**< 0.0001**	−46.43%	−0.13 (− 0.21;-0.05)	**0.0007**	−64.29%	− 0.19 (− 0.27;-0.11)	**< 0.0001**	10.87%	0.06 (− 0.02;0.14)	0.2
Seeds and dried fruits	0.22 ± 0.62	0.22 ± 0.59	0.18 ± 0.71	0.2	−22.22%	− 0.04 (− 0.11;0.02)	0.3	− 22.22%	− 0.04 (− 0.10;0.02)	0.2	0%	0.00 (− 0.07;0.06)	1.0
Starches	2.33 ± 2.57	2.53 ± 2.13	1.73 ± 1.97	**< 0.0001**	−34.68%	−0.60 (− 0.82;-0.38)	**< 0.0001**	−46.24%	−0.80 (−1.02;-0.59)	**< 0.0001**	7.91%	0.20 (− 0.02;0.42)	0.1
**Milk products and eggs**	3.00 ± 3.18	3.33 ± 3.53	2.41 ± 3.45	**< 0.0001**	−24.48%	−0.59 (− 0.93;-0.26)	**< 0.0001**	−38.17%	− 0.93 (−1.26;-0.60)	**< 0.0001**	9.91%	0.33 (0.00;0.66)	**0.05**
**Cheeses**	1.41 ± 2.38	1.30 ± 1.80	0.86 ± 1.46	**< 0.0001**	−63.95%	−0.55 (− 0.73;-0.36)	**< 0.0001**	−51.16%	− 0.43 (− 0.62;-0.25)	**< 0.0001**	−8.46%	− 0.11 (− 0.30;0.08)	0.4
**Meat and fish**													
Raw meat	1.60 ± 2.74	1.74 ± 2.58	1.53 ± 2.10	0.09	−4.58%	−0.07 (− 0.31;0.17)	0.8	−13.73%	− 0.21 (− 0.45;0.03)	0.08	8.05%	0.14 (− 0.10;0.38)	0.3
Ultra-processed meat	1.05 ± 1.60	1.01 ± 1.46	0.87 ± 1.33	**0.009**	−20.69%	−0.18 (− 0.32;-0.03)	**0.009**	−16.09%	− 0.14 (− 0.28;0.00)	0.06	−3.96%	− 0.04 (− 0.18;0.11)	0.8
Delicatessen	0.47 ± 0.98	0.47 ± 0.90	0.30 ± 0.70	**< 0.0001**	−56.67%	−0.17 (− 0.25;-0.08)	**< 0.0001**	−56.67%	−0.17 (− 0.25;-0.08)	**< 0.0001**	0%	0.00 (− 0.08;0.09)	1.0
Raw fish	0.56 ± 1.34	0.52 ± 0.98	0.37 ± 0.87	**< 0.0001**	−51.35%	−0.19 (− 0.29;-0.08)	**0.0001**	−40.54%	− 0.15 (− 0.25;-0.05)	**0.0008**	−7.69%	− 0.04 (− 0.14;0.07)	0.9
Processed fish	0.37 ± 0.85	0.32 ± 0.72	0.25 ± 0.62	**0.0006**	−48%	−0.12 (− 0.19;-0.05)	**0.0004**	−28%	− 0.07 (− 0.14;0.00)	**0.04**	−15.63%	−0.05 (− 0.12;0.02)	0.3
Ultra-processed fish	0.38 ± 1.32	0.37 ± 0.85	0.26 ± 0.70	**0.007**	−46.15%	−0.12 (− 0.21;-0.02)	**0.02**	− 42.31%	− 0.11 (− 0.2;-0.01)	**0.02**	− 2.7%	−0.01 (− 0.11;0.09)	1.0
Seafood products	0.08 ± 0.32	0.13 ± 0.43	0.09 ± 0.36	**0.003**	11.11%	0.01 (−0.03;0.05)	0.8	− 44.44%	− 0.04 (− 0.08;0.00)	**0.02**	38.46%	0.05 (0.01;0.09)	**0.004**
**Sugary products**													
Sweet biscuits	0.81 ± 1.41	0.95 ± 1.68	0.66 ± 1.34	**< 0.0001**	−22.73%	−0.15 (− 0.29;0.00)	**0.04**	−43.94%	− 0.28 (− 0.43;-0.14)	**< 0.0001**	14.74%	0.14 (0.00;0.28)	0.09
Confectionery and spreads	1.23 ± 2.05	1.42 ± 2.07	0.93 ± 1.68	**< 0.0001**	−32.26%	− 0.3 (− 0.49;-0.11)	**0.0006**	−52.69%	− 0.49 (− 0.68;-0.31)	**< 0.0001**	13.38%	0.19 (0.00;0.38)	**0.03**
Pie dough	0.22 ± 0.63	0.22 ± 0.65	0.13 ± 0.49	**0.0002**	−69.23%	−0.09 (− 0.15;-0.03)	**0.001**	− 69.23%	− 0.09 (− 0.15;-0.03)	**0.0007**	0%	0.00 (− 0.06;0.06)	1.0
Cream desserts	0.36 ± 0.87	0.38 ± 0.89	0.34 ± 0.79	0.6	−5.88%	−0.01 (− 0.10;0.07)	0.9	−11.76%	− 0.04 (− 0.12;0.05)	0.5	5.26%	0.02 (− 0.06;0.10)	0.8
Bread and pastries	1.01 ± 1.54	1.09 ± 1.50	0.75 ± 1.19	**< 0.0001**	−34.67%	−0.26 (− 0.4;-0.12)	**< 0.0001**	−45.33%	−0.34 (− 0.48;-0.20)	**< 0.0001**	7.34%	0.08 (− 0.06;0.22)	0.5
Breakfast cereals	0.32 ± 0.79	0.38 ± 0.78	0.25 ± 0.60	**0.0002**	−28%	−0.06 (− 0.13;0.01)	0.07	−52%	− 0.13 (− 0.19;-0.06)	**0.0001**	15.79%	0.06 (− 0.01;0.13)	0.2
Ice creams	0.20 ± 0.70	0.20 ± 0.58	0.15 ± 0.46	**0.04**	−33.33%	−0.05 (− 0.11;0.00)	0.08	− 33.33%	− 0.05 (− 0.11;0.00)	0.06	0%	0.00 (− 0.06;0.05)	1.0
**Salty products**													
Composite dishes, pizzas and sandwiches	0.74 ± 1.55	0.81 ± 1.72	0.64 ± 1.64	**0.04**	−15.63%	−0.10 (− 0.27;0.06)	0.2	−26.56%	− 0.17 (− 0.33;-0.01)	**0.03**	8.64%	0.07 (− 0.09;0.23)	0.7
Savoury aperitif products	0.41 ± 1.01	0.48 ± 1.12	0.31 ± 0.90	**0.0002**	−32.26%	−0.10 (− 0.20;0.00)	0.06	−54.84%	− 0.17 (− 0.27;-0.07)	**0.0001**	14.58%	0.08 (− 0.02;0.17)	0.2
Salades	0.02 ± 0.16	0.04 ± 0.31	0.03 ± 0.25	0.2	33.33%	0.01 (−0.02;0.03)	0.7	−33.33%	−0.01 (− 0.03;0.01)	0.6	50%	0.02 (−0.01;0.04)	0.2
Soups	0.10 ± 0.43	0.11 ± 0.38	0.06 ± 0.29	**0.0007**	−66.67%	−0.05 (− 0.08;-0.01)	**0.01**	−83.33%	− 0.06 (− 0.09;-0.02)	**0.001**	9.09%	0.01 (− 0.03;0.05)	0.8
Vegetable soups	0.19 ± 0.82	0.22 ± 0.58	0.15 ± 0.50	**0.03**	−26.67%	−0.05 (− 0.11;0.02)	0.2	−46.67%	− 0.07 (− 0.13;-0.01)	**0.03**	13.64%	0.02 (− 0.04;0.09)	0.7
**Sauces and condiments**	1.16 ± 2.03	1.21 ± 1.99	0.80 ± 1.57	**< 0.0001**	−45%	−0.36 (− 0.54;-0.18)	**< 0.0001**	−51.25%	− 0.41 (− 0.59;-0.23)	**< 0.0001**	4.13%	0.05 (− 0.13;0.23)	0.7
**Fats and oils**	0.90 ± 1.36	0.97 ± 1.16	0.73 ± 1.09	**< 0.0001**	−23.29%	−0.17 (− 0.29;-0.05)	**0.003**	−32.88%	− 0.24 (− 0.35;-0.13)	**< 0.0001**	7.22%	0.07 (− 0.04;0.19)	0.2
**Beverages**													
Waters	1.79 ± 3.30	1.84 ± 3.22	1.58 ± 3.13	0.1	−13.29%	−0.22 (− 0.54;0.10)	0.3	−16.46%	− 0.27 (− 0.58;0.05)	0.1	2.72%	0.05 (− 0.27;0.37)	0.9
Fruits juices	0.65 ± 1.60	0.65 ± 1.17	0.58 ± 1.01	**0.3**	−12.07%	−0.08 (− 0.20;0.05)	0.3	− 12.07%	−0.07 (− 0.20;0.05)	0.4	0%	0.00 (− 0.13;0.12)	1.0
Syrups and sweetened beverages	0.39 ± 1.17	0.46 ± 1.26	0.33 ± 0.90	**0.03**	−18.18%	−0.06 (− 0.17;0.05)	0.4	−39.39%	− 0.13 (− 0.24;-0.02)	**0.02**	15.22%	0.07 (− 0.04;0.18)	0.4
Tea, hot chocolate and coffea	0.68 ± 0.98	0.87 ± 1.06	0.55 ± 0.95	**< 0.0001**	−23.64%	−0.13 (− 0.23;-0.04)	**0.004**	−58.18%	− 0.33 (− 0.42;-0.23)	**< 0.0001**	21.84%	0.19 (0.09;0.29)	**< 0.0001**

**Table 4:** Proportions of food products in the different categories by trial arm in the overall sample (*N* = 3382 participants overall)

Food category	Proportions in percentage				Nutri-Score vs no label		Nutri-Score vs Reference Intakes		Reference Intakes vs no label	
	No label	Reference Intakes	Nutri-Score	P-value	Difference [95%CI]	P-value	Difference [95%CI]	P-value	Difference [95%CI]	P-value
**Fruits, vegetables, legumes and nuts**										
Raw fruits^a^	6.52 ± 6.62	6.25 ± 6.96	12.72 ± 19.19	**< 0.0001**	6.20 (4.98;7.43)	**< 0.0001**	6.47 (5.27;7.68)	**< 0.0001**	−0.27 (−1.49;0.95)	0.9
Processed fruits^a^	0.49 ± 1.55	0.64 ± 1.89	1.96 ± 7.89	**< 0.0001**	1.47 (0.99;1.94)	**< 0.0001**	1.31 (0.85;1.78)	**< 0.0001**	0.15 (− 0.32;0.63)	0.8
Ultra-processed fruits^a^	0.54 ± 1.68	0.53 ± 1.61	0.56 ± 1.89	0.9	0.03 (−0.15;0.20)	0.9	0.03 (−0.14;0.20)	0.9	−0.01 (− 0.18;0.16)	1.0
Raw vegetables^a^	9.23 ± 9.66	9.06 ± 8.82	7.18 ± 10.99	**< 0.0001**	−2.05 (−3.04;-1.07)	**< 0.0001**	−1.88 (− 2.84;-0.91)	**< 0.0001**	− 0.18 (− 1.15;0.8)	0.9
Processed vegetables^a^	1.88 ± 3.43	1.94 ± 3.38	1.82 ± 3.66	0.8	−0.05 (− 0.40;0.29)	0.9	− 0.11 (− 0.45;0.23)	0.8	0.06 (− 0.28;0.40)	1.0
Ultra-processed vegetables^a^	1.99 ± 5.14	1.87 ± 3.16	1.75 ± 3.66	0.4	−0.24 (− 0.65;0.16)	0.3	− 0.12 (− 0.52;0.27)	0.8	−0.12 (− 0.52;0.28)	0.7
Legumes	1.26 ± 3.06	1.42 ± 3.12	0.91 ± 2.72	**< 0.0001**	− 0.35 (− 0.64;-0.06)	**0.02**	− 0.52 (− 0.80;-0.23)	**< 0.0001**	0.17 (− 0.13;0.46)	0.3
Seeds and dried fruits	0.65 ± 2.32	0.56 ± 1.81	0.46 ± 1.74	0.08	−0.19 (− 0.39;0.00)	0.06	− 0.11 (− 0.30;0.08)	0.3	−0.08 (− 0.28;0.11)	0.7
Starches	8.37 ± 7.65	9.44 ± 9.31	6.80 ± 7.30	**< 0.0001**	−1.56 (−2.36;-0.77)	**< 0.0001**	− 2.64 (− 3.43;-1.86)	**< 0.0001**	1.08 (0.29;1.87)	**0.008**
**Milk products and eggs**	11.37 ± 10.99	11.74 ± 10.09	9.37 ± 10.24	**< 0.0001**	−1.99 (−3.03;-0.95)	**< 0.0001**	− 2.37 (− 3.39;-1.35)	**< 0.0001**	0.38 (− 0.65;1.41)	0.7
**Cheeses**	4.60 ± 5.84	3.81 ± 5.84	3.07 ± 5.14	**< 0.0001**	−1.53 (−2.09;-0.98)	**< 0.0001**	−0.75 (− 1.29;-0.20)	**0.004**	− 0.79 (− 1.34;-0.23)	**0.003**
**Meat and fish**										
Raw meat^a^	5.16 ± 6.51	5.33 ± 6.22	8.35 ± 12.5	**< 0.0001**	3.19 (2.31;4.06)	**< 0.0001**	3.02 (2.16;3.88)	**< 0.0001**	0.17 (− 0.70;1.04)	0.8
Ultra-processed meat^a^	3.91 ± 6.34	3.23 ± 4.59	4.66 ± 10.02	**< 0.0001**	0.75 (0.02;1.48)	**0.05**	1.43 (0.71;2.14)	**< 0.0001**	−0.68 (−1.40;0.04)	0.06
Delicatessen	1.65 ± 4.47	1.38 ± 2.77	0.97 ± 2.57	**< 0.0001**	−0.68 (−1.01;-0.34)	**< 0.0001**	− 0.4 (− 0.73;-0.07)	**0.01**	−0.27 (− 0.61;0.06)	0.1
Raw fish^a^	1.84 ± 4.16	1.60 ± 4.14	1.59 ± 4.67	0.4	−0.25 (− 0.67;0.17)	0.4	− 0.01 (− 0.43;0.40)	1.0	− 0.24 (− 0.66;0.18)	0.5
Processed fish^a^	1.84 ± 7.38	0.81 ± 1.96	0.76 ± 1.96	**< 0.0001**	−1.08 (− 1.53;-0.63)	**< 0.0001**	−0.05 (− 0.49;0.39)	1.0	−1.03 (− 1.47;-0.58)	**< 0.0001**
Ultra-processed fish^a^	1.06 ± 3.93	1.03 ± 2.58	0.77 ± 2.13	**0.04**	−0.29 (− 0.59;0.00)	0.06	− 0.26 (− 0.55;0.03)	0.07	−0.03 (− 0.32;0.26)	1.0
Seafood products	0.27 ± 1.83	0.37 ± 1.87	0.38 ± 2.06	0.3	0.11 (−0.08;0.30)	0.4	0.01 (−0.18;0.20)	1.0	0.10 (− 0.09;0.29)	0.4
**Sugary products**										
Sweet biscuits	3.22 ± 7.05	3.49 ± 8.33	2.25 ± 4.33	**< 0.0001**	−0.98 (−1.65;-0.31)	**0.001**	−1.24 (− 1.90;-0.58)	**< 0.0001**	0.27 (− 0.4;0.93)	0.7
Confectionery and spreads	3.57 ± 5.16	3.80 ± 4.53	3.07 ± 5.87	**0.002**	−0.51 (−1.02;0.01)	0.06	−0.73 (−1.24;-0.22)	**0.002**	0.22 (− 0.29;0.74)	0.5
Pie dough	0.53 ± 1.61	0.56 ± 1.69	0.33 ± 1.32	**0.0004**	−0.21 (− 0.36;-0.05)	**0.005**	− 0.23 (− 0.39;-0.08)	**0.0008**	0.03 (− 0.12;0.18)	0.9
Cream desserts	1.26 ± 4.45	1.11 ± 3.85	2.07 ± 7.03	**< 0.0001**	0.81 (0.28;1.33)	**0.001**	0.96 (0.44;1.48)	**< 0.0001**	−0.15 (− 0.68;0.37)	0.8
Bread and pastries	3.56 ± 5.72	3.51 ± 4.66	3.42 ± 6.70	0.8	−0.14 (− 0.70;0.43)	0.8	− 0.10 (− 0.65;0.46)	0.9	−0.04 (− 0.6;0.52)	0.9
Breakfast cereals	1.11 ± 2.57	1.30 ± 3.95	0.99 ± 2.48	0.06	−0.11 (− 0.41;0.19)	0.6	− 0.31 (− 0.61;-0.01)	0.05	0.20 (− 0.10;0.50)	0.4
Ice creams	0.53 ± 1.80	0.51 ± 1.88	0.46 ± 1.71	0.7	−0.07 (− 0.25;0.11)	0.7	− 0.05 (− 0.22;0.13)	0.8	−0.02 (− 0.2;0.15)	1.0
**Salty products**										
Composite dishes, pizzas and sandwiches	2.68 ± 6.16	2.53 ± 5.65	2.15 ± 5.11	0.06	−0.53 (−1.09;0.02)	0.05	−0.38 (− 0.92;0.17)	0.3	− 0.16 (− 0.71;0.40)	0.7
Savoury aperitif products	1.21 ± 4.28	1.52 ± 5.48	0.87 ± 2.62	**0.002**	−0.34 (− 0.77;0.08)	0.1	− 0.65 (−1.07;-0.23)	**0.001**	0.30 (− 0.12;0.73)	0.2
Salades	0.06 ± 0.44	0.09 ± 0.56	0.14 ± 1.51	0.2	0.08 (−0.02;0.17)	0.1	0.05 (− 0.04;0.14)	0.4	0.03 (− 0.07;0.12)	0.8
Soups	0.33 ± 1.41	0.52 ± 3.91	0.21 ± 1.44	**0.01**	−0.12 (− 0.37;0.14)	0.5	− 0.31 (− 0.56;-0.06)	**0.01**	0.19 (− 0.06;0.45)	0.2
Vegetable soups	0.70 ± 3.75	0.74 ± 2.41	0.50 ± 1.82	0.08	−0.20 (− 0.48;0.07)	0.2	− 0.24 (− 0.51;0.03)	0.1	0.04 (− 0.23;0.31)	1.0
**Sauces and condiments**	3.16 ± 5.18	2.98 ± 4.31	2.64 ± 6.42	0.06	−0.53 (−1.05;0.00)	0.06	−0.34 (− 0.86;0.18)	0.2	−0.18 (− 0.71;0.34)	0.8
**Fats and oils**	2.77 ± 4.51	2.94 ± 4.45	3.03 ± 6.39	0.4	0.26 (−0.24;0.77)	0.4	0.09 (−0.41;0.59)	0.9	0.17 (−0.33;0.68)	0.6
**Beverages**										
Waters	6.51 ± 12.75	5.79 ± 9.78	6.72 ± 11.75	0.1	0.21 (−0.93;1.35)	0.9	0.93 (−0.19;2.05)	0.1	−0.72 (−1.85;0.41)	0.3
Fruits juices	2.52 ± 5.07	2.46 ± 5.60	3.61 ± 8.43	**< 0.0001**	1.09 (0.45;1.73)	**0.0003**	1.16 (0.52;1.79)	**< 0.0001**	−0.07 (− 0.70;0.57)	0.9
Syrups and sweetened beverages	1.40 ± 4.22	1.35 ± 3.42	1.64 ± 5.67	0.2	0.25 (−0.20;0.69)	0.4	0.29 (−0.15;0.73)	0.2	−0.04 (− 0.49;0.40)	0.9
Tea, hot chocolate and coffea	2.24 ± 3.49	3.77 ± 7.84	1.82 ± 3.26	**< 0.0001**	−0.42 (− 0.95;0.11)	0.2	−1.95 (− 2.47;-1.43)	**< 0.0001**	1.53 (1.00;2.06)	**< 0.0001**

^a^ These sub-categories correspond to the food categories for which a distinction was made by the degree of processing. Raw foods corresponded to NOVA 1 group, processed foods to NOVA 2 group and ultra-processed foods to NOVA 4 group. No product from NOVA 3 group was found for the ‘Meat’ category, resulting in only two sub-categories

## Discussion

The present findings provide insights on the effect of the Nutri-Score on the shopping cart composition, regarding the type of food products (pre-packed versus unpacked) and the degree of processing. Results have shown that overall, participants – students, low-income individuals and subjects suffering from cardiometabolic diseases – simulating purchases with the Nutri-Score affixed on pre-packed foods were more likely to have higher proportions of unpacked products – especially unprocessed fruits and meat – in their shopping cart compared to the two other arms. This finding would be related to a decrease of purchasing intentions for manufactured processed or ultra-processed products – especially those classified as ‘C’ and ‘D’.

These specific results could therefore explain the results of previous analyses on these three randomized trials, where the Nutri-Score was observed to improve the overall nutritional quality of purchasing intentions compared to the two other arms or the RIs only, with in particular a decrease of the shopping carts contents in calories and SFA [21]. While the Nutri-Score takes into account the food nutritional composition only, the present findings suggest that this FoPL would have an effect on the type of purchased foods, with less ultra-processed foods which have been found to be often of lower nutritional quality [26–29]. This effect of the Nutri-Score, which might encourage consumers towards unprocessed fruits and meats, and less manufactured processed products and notably less ultra-processed foods such as for fruits and fishes, is in lines with dietary guidelines. Indeed, French public health messages are promoting the consumption of unprocessed foods since 2019, and a decrease in the consumption of ultra-processed foods [20], as they have been observed to increase the risk of diseases [30–37]. Several assumptions may explain the effect of the Nutri-Score on processed foods purchases. First, this FoPL could raise awareness of consumers overall on the healthiness of foods encouraging healthier purchasing behavior. Second, the application of a worse Nutri-Score on foods (‘C’ or ‘D’) could discourage consumers and encourage them towards some raw foods, known as healthy products such as fruits. However, the Nutri-Score seemed to decrease purchases in raw fish and especially in vegetables, for which no clear hypothesis is available. Nevertheless, it could be partly related to the fact that participants exposed to the Nutri-Score in the study would have balanced their fruits and vegetables budget by purchasing more raw fruits but less raw vegetables, without any change on processed or ultra-processed vegetables – raw products being more expensive.

The trials were conducted on an experimental online supermarket, similar to real online grocery shopping conditions, with ecological validity given the recent increase of online purchases [38]. Moreover, a large sample of food products with real packaging, from different food categories, brands, and degree of processing were included. However, some limitations should be acknowledged. First, high rates of non-respondents (between 36 and 50% depending on the trial) were observed in the three trials, which could have led to a lower statistical power preventing us from detecting some potential small differences in analyses by population. Second, the diversity of the food offer in the online supermarket remained somewhat limited, and participants could have chosen some foods they would not buy in real-life situation. In particular, offer for fresh products is usually limited in online supermarkets compared to physical stores. In addition, the trials investigated purchasing intentions rather than actual food purchases. Nevertheless, it has been suggested that virtual purchasing behaviours of consumers would be good predictors of real behaviours [39]. Furthermore, the food offer was not selected for this specific purpose – to investigate the effect on the nature of products – resulting in unbalanced distributions of products across food categories and processing degree. Consequently, the distinction of products across NOVA groups could be made on four categories only.

Beyond elements pertaining to the environment of the experiments, the population that was included in the various studies may differ from the general population; our results may not be directly extrapolated to the overall population. Finally, results should be interpreted with caution given that they correspond to post-hoc analyses. Complementary studies should be performed to specifically investigate this issue.

The present analyses provide new insights regarding the positive impact of the Nutri-Score on food purchases which would discourage the purchase of pre-packed processed products. Nevertheless, this positive effect did not impact all food categories and specific communications could therefore be made. These elements are particularly important in the specific European context, where a growing number of countries are implementing this FoPL and discussions currently ongoing labeling harmonization at European level.

## Supplementary Information

**Additional file 1: Supplemental Figure 1.** Flowcharts of the three randomized controlled trials.

**Additional file 2: Supplemental Figure 2.** Images of the Nutri-Score and the Reference Intakes labels used in the trials.

## Data Availability

All data supporting the findings of this study are included in the present article or the supplemental material.

## Abbreviations

FoPL: Front-of-Pack Label
FSAm-NPS: Food Standards Agency modified - Nutrient Profiling system
RIs: Reference Intakes
SFA: Saturated Fatty Acids
