# Supplementary material for: The impact of the Nutri-Score front-of-pack nutrition label on purchasing intentions of unprocessed and processed foods: post-hoc analyses from three randomized controlled trials
Source: Int J Behav Nutr Phys Act. 2021 Mar 17;18:38. doi: 10.1186/s12966-021-01108-9 (PMC7968175; doi:10.1186/s12966-021-01108-9)
Supplement: Supplementary file 1 — Additional file 1: Supplemental Figure 1. Flowcharts of the three randomized controlled trials. [file 12966_2021_1108_MOESM1_ESM.docx]

3507 students assessed for eligibility

970 assigned to Nutri-Score

977 assigned to no label

960 assigned to RIs

2907 students randomized

600 ineligible because they did not meet inclusion criteria (age, profession, grocery shopping)

623 included in analyses

592 included in analyses

651 included in analyses

919 working adults with low incomes assessed for eligibility

173 assigned to Nutri-Score

174 assigned to no label

177 assigned to RIs

524 working adults randomized

395 ineligibles because they did not meet inclusion criteria (age, profession, monthly income, grocery shopping)

115 included in analyses

105 included in analyses

116 included in analyses

394 included in analyses

394 included in analyses

392 included in analyses

3,728 individuals with chronic diseases assessed for eligibility

2,431 individuals with chronic diseases randomized

1,297 ineligible because they did not meet inclusion criteria (age, chronic disease, grocery shopping)

814 assigned to Nutri-Score

814 assigned to no label

803 assigned to RIs

**Supplemental Figure 1. Flowcharts of the three randomized controlled trials**
