# Supplementary material for: The impact of the Nutri-Score front-of-pack nutrition label on purchasing intentions of unprocessed and processed foods: post-hoc analyses from three randomized controlled trials
Source: Int J Behav Nutr Phys Act. 2021 Mar 17;18:38. doi: 10.1186/s12966-021-01108-9 (PMC7968175; doi:10.1186/s12966-021-01108-9)
Supplement: Supplementary file 2 — Additional file 2: Supplemental Figure 2. Images of the Nutri-Score and the Reference Intakes labels used in the trials. [file 12966_2021_1108_MOESM2_ESM.docx]

**
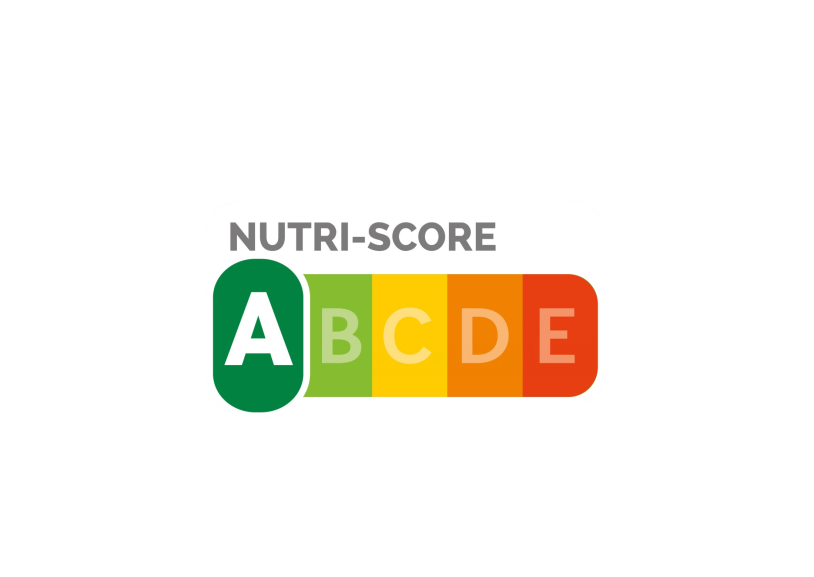
**

**
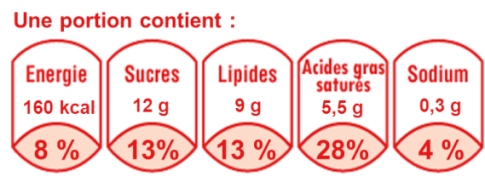
**

**Supplemental Figure 2. Images of the Nutri-Score and the Reference Intakes labels used in the trials**
